# The Cost of Provisions in Institutions.—XI

**Published:** 1904-07-09

**Authors:** 


### HOSPITAL ADMINISTRATION.

### CONSTRUCTION AND ECONOMICS.

### THE COST OF PROVISIONS IN INSTITUTIONS.—XI.

By the Author of "Hospital Expenditure—The Commissariat."

(*Continued from page* 230.)

ST. MARY'S HOSPITAL.—(Available Beds, 281).

The cost of provisions per bed at this hospital during the last five years is as follows:—

Table I.

| Year | Average cost per occupied bed | Average of occupied beds |
|---|---|---|
| | £ | |
| 1902 ... ... ... | 31 | 248 |
| 1901 ... ... ... | 29½ | 250 |
| 1900 ... ... ... | 30¼ | 254 |
| 1899 ... ... ... | 30¾ | 198 |
| 1898 ... ... ... | 29¼ | 245 |

Bearing in mind that the average cost of provisions per bed includes the board of all the inmates of the hospital, it will be seen that the fluctuations in cost from year to year are very slight.

Eliminating the cost of the patient, which the admirable system of accounts in use at St. Mary's renders possible, we have a more accurate estimate of the cost of maintaining the institution based on the average of available beds, the cost of provisions for the more permanent elements of the household being divided by 281.

Table II.

| Year | Number of occupied beds out of 281 available | Total cost of provisions | Cost of patients at £16 a year | Average cost of provisions, exclusive of patients | |
|---|---|---|---|---|---|
| | | | | Per available bed | Per occupied bed |
| | | £ | £ | £ | £ |
| 1902 | 248 | 7,723 | 3,968 | 13½ | 15 |
| 1901 | 250 | 7,389 | 4,000 | 12 | 13½ |
| 1900 | 254 | 7,689 | 3,625 | 12¾ | 14¼ |
| 1899 | 198 | 6,094 | 3,168 | 10½ | 14¾ |
| 1898 | 245 | 7,180 | 3,920 | 11½ | 13¼ |

An analysis of those boarded in the hospital other than patients is given below:—

Table III.

| Personal | Number | Average of available beds to each | Average of occupied beds to each |
|---|---|---|---|
| Medical and other officers ... ... | 19 | 15 | 13 |
| Nursing staff ... | 98 | 2·8 | 2·5 |
| Wardmaids and female servants, including laundry maids ... ... | 36 | 8 | 7 |
| Male servants ... | 13 | — | — |
| Total ... ... | 166 | 1·7 | 1·4 |

Those boarded other than patients are 40 per cent. of all the inmates.

Referring to Table II. it will be seen that the figures quoted are of the nature of an estimate, based on a calculation that the cost of the patient's food is 10½d. a day or £16 a year. In these and similar estimates which we have worked out it has been assumed that the average cost of the patient per head varies very little from year to year. We are unexpectedly able to verify the conclusions arrived at in this particular by the courtesy of the secretary of St. Mary's Hospital, who has kindly furnished us with details of a week's expenditure on food in the hospital, May 8th to 14th, 1904. This valuable record, which we are permitted to quote, shows the actual consumption of food under 36 headings. It is based on the weekly analytical account of provisions subdivided into two classes:—1. Patients; 2. Household; (*a*) Officers; (*b*) Nurses; (*c*) Servants.

It must be noted that, in order to provide for the classification of this record into patients and household, the stores required are ordered and invoiced for each class separately. This ensures accuracy which the most painstaking estimate cannot guarantee. The figures for the given week work out as follows:—

Cost of patients .................. £80 10s. 11d.

Cost of household.................. £69 10s. 10d.

The daily average of occupied beds during this week was 264·4; the cost per patient per day was therefore 10·4d. The cost of the household, including 18 officers, 97 nurses, and 53 servants amounted to an average per head, per day, of 1s. 2·1d.

The figures in Table II. for 1902 coincide remarkably with this specimen week. The total cost of provisions in that year was £7,723. Deducting the cost of an average of 248 patients at 10½d. a day (£3,968), there remains £3,755 for the board of the then daily average of 166 persons other than patients, the cost of whom works out at 1s. 3d. per head per day.

Bearing in mind the inevitable small variations in actual cost of one week with another, it will be seen that the expenditure of the year 1902 shows a remarkable accord with the expenditure of the selected week in 1904.

The expenditure, however, as analysed in the sheet before us, is subdivided, so that the cost of officers, nurses, and servants, is clearly to be ascertained, and is as follows:—

Cost per officer per day 2s. 1d. (very nearly), or rather under £40 a year.

Cost per nurse per day 1s. 5d. (nearly), or £26 a year.

Cost per servant per day 6d. (nearly), or £9 a year.

In estimating the cost per individual of various articles enumerated under the heading of provisions in the accounts for 1902, numbers only have been taken into account. But as in all the hospitals of which we have thus analysed the accounts, the same elements are to be found, and on the whole in fairly constant proportions, a comparison of the cost per bed and per head of these items is not uninstructive.

Daily average of patients ..........................248
Daily average of non-patients ......................166
Daily average boarded in hospital ..................414

Table IV.

| Provisions | Total cost | Cost per head all inmates | Cost per occupied bed |
|---|---|---|---|
| | £ | £ s. | £ s. |
| Meat ... ... ... | 2,138 | 5 3 | 8 12 |
| Fish, poultry, etc ... | 1,072 | 2 10 | 4 6 |
| Butter, cheese, etc. ... | 937 | 2 5 | 3 15 |
| Eggs ... ... ... | 689 | 1 13 | 2 15 |
| Milk ... ... ... | 1,199 | 2 17 | 4 16 |
| Bread, flour, etc. ... | 469 | 1 2 | 1 17 |
| Grocery ... ... | 664 | 1 12 | 2 13 |
| Vegetables ... ... | 324 | 15 | 1 6 |
| Malt liquor ... ... | 227 | 10 | 18 |
| Ice and mineral waters ... ... | 54 | — | 4* |
| Wine and spirits ... | 253 | — | 1 0* |

* Patients only

The data supplied by the provision-book throw considerable light on the proportionate expense of patients and household for these items. The cost of meat for the patients in the given week was £7 8s. 8d., out of a total of £27 2s. 8d. for the whole hospital. This makes the cost per patient per week for meat 6·7d., but to this must be added £2 14s. for meat for beef tea, which would add an extra 1½d. to the average weekly cost of the patient under this heading.

If this specimen week were to be regarded as an average for the year, the yearly cost of the patient for meat works out at £1 16s., and the yearly cost of each member of the household for the same item, at £5, which it will be seen, amounts to considerably more than the actual yearly expenditure per head on all the inmates taken together. But as all housekeepers know, expenditure on particular items must necessarily vary from week to week.

The average quantity of uncooked meat required per head per week was for the patients 1¼ lb., for the household nearly 5 lb.

The quantity of milk required averaged just over 3 pints per week or rather less than half-a-pint per day for the household, milk evidently not being used as a beverage. For the patients, the amount averaged 19 pints a week, or about 2¾ pints a day each. The milk at St. Mary's is frequently tested, and every precaution is taken to ensure that its quality is kept up to the required standard.

We may add in conclusion that the cost of boarding the laundry maids is included in this hospital under the heading of provisions, and not, as in some others, under the head of washing.

